# *In Vitro* HIV-1 Selective Integration into the Target Sequence and Decoy-Effect of the Modified Sequence

**DOI:** 10.1371/journal.pone.0013841

**Published:** 2010-11-04

**Authors:** Tatsuaki Tsuruyama, Tonau Nakai, Takuya Hiratsuka, Guang Jin, Takuro Nakamura, Kenichi Yoshikawa

**Affiliations:** 1 Department of Forensic Medicine and Molecular Pathology, Graduate School of Medicine, Kyoto University, Kyoto, Kyoto Prefecture, Japan; 2 Department of Physics, Graduate School of Science, Kyoto University, Kyoto, Kyoto Prefecture, Japan; 3 Laboratory of Pathology, Noe-saiseikai Hospital, Osaka, Osaka Prefecture, Japan; 4 Division of Carcinogenesis, The Cancer Institute, Japanese Foundation for Cancer Research, Koto-ku, Tokyo, Japan; Tsinghua University, China

## Abstract

Although there have been a few reports that the HIV-1 genome can be selectively integrated into the genomic DNA of cultured host cell, the biochemistry of integration selectivity has not been fully understood. We modified the *in vitro* integration reaction protocol and developed a reaction system with higher efficiency. We used a substrate repeat, 5′-(GTCCCTTCC*CAGT* )*_n_*(*ACTG* GGAAGGGAC)*_n_*-3′, and a modified sequence DNA ligated into a circular plasmid. *CAGT* and *ACTG* (shown in italics in the above sequence) in the repeat units originated from the HIV-1 proviral genome ends. Following the incubation of the HIV-1 genome end cDNA and recombinant integrase for the formation of the pre-integration (PI) complex, substrate DNA was reacted with this complex. It was confirmed that the integration selectively occurred in the middle segment of the repeat sequence. In addition, integration frequency and selectivity were positively correlated with repeat number *n*. On the other hand, both frequency and selectivity decreased markedly when using sequences with deletion of *CAGT* in the middle position of the original target sequence. Moreover, on incubation with the deleted DNAs and original sequence, the integration efficiency and selectivity for the original target sequence were significantly reduced, which indicated interference effects by the deleted sequence DNAs. Efficiency and selectivity were also found to vary discontinuously with changes in manganese dichloride concentration in the reaction buffer, probably due to its influence on the secondary structure of substrate DNA. Finally, integrase was found to form oligomers on the binding site and substrate DNA formed a loop-like structure. In conclusion, there is a considerable selectivity in HIV-integration into the specified sequence; however, similar DNA sequences can interfere with the integration process, and it is therefore difficult for *in vivo* integration to occur selectively in the actual host genome DNA.

## Introduction

Integration into the host cell genome is an important process in the life cycle of HIV-1. Once integrated, the retroviral genome becomes a stable part of the host genome, and is subsequently duplicated as a provirus during host cell division. The integration reaction is catalyzed by integrase, which is encoded in the retroviral genome. Recent therapeutic developments to combat AIDS have focused on integrase inhibitors such as Raltegravir in order to reduce side effects[1], [2] and second-generation HIV-1 integrase inhibitoers have been developed [3]. The development of IN inhibitors aims to combat viral resistance to earlier drug classes. On the other hand, understanding of the molecular mechanisms of integration is insufficient, although the translocation process of the pre-integration complex in the nucleus and integration selectivity are being extensively studied. Schroeder et al. performed a genome-wide screening of integration sites using a cell culture system with HIV-1 infection and identified integration sites throughout whole chromosome [4]. Following statistical analysis, they reported that integration preferentially occurred at transcriptionally active genes, and similar data on murine leukemia retroviral integration were reported [5]–[7]. Several mechanisms have been proposed that chromatin accessibility influence the integration site selection [8]. Recent data provide evidence that selective integration can occur via a tethering mechanism through the recruitment of the lentiviral integrase by the cellular LEDGF/p75 protein, which have been recognized as the target of anti-integration therapy [2].

On the other hand, Yoshinaga et al. reported an *in vitro* integration assay method and confirmed the terminal oligonucleotide motif at the HIV-1 genome end as an integration signal sequence motif (RSS). The RSS consisted of heptamer 5′-AGCAGT-3′, and replacement of one nucleotide in RSS significantly suppressed the integration of HIV-1 [9]. We believe that HIV-1 integrase has the potential to select the integration site because RSS is expected to favor its complementary sequence in the target sequence. Their study suggested that HIV-1 integrase has the potential to select the integration site. In the present study, we modified their method in order to identify the precise HIV-1 integration sites and improve the efficiency of *in vitro* integration.

In this study, we used a repeat DNA sequence, 5′-(GT-oligo-puCAGT) *_6_*(ACTG oligopy-AC) *_6_*-3′, with a repeat element identical to the sequence at the HIV-1 3′-terminus, and included the CATG integration signal sequence. In the previous methodology, such specified sequence motif DNAs have not been used. Thus, we applied our modified protocol using a repeat sequence for efficiency and selectivity of the *in vitro* integration.

## Materials and Methods

### *In vitro* integration assay

**Figure 1:**
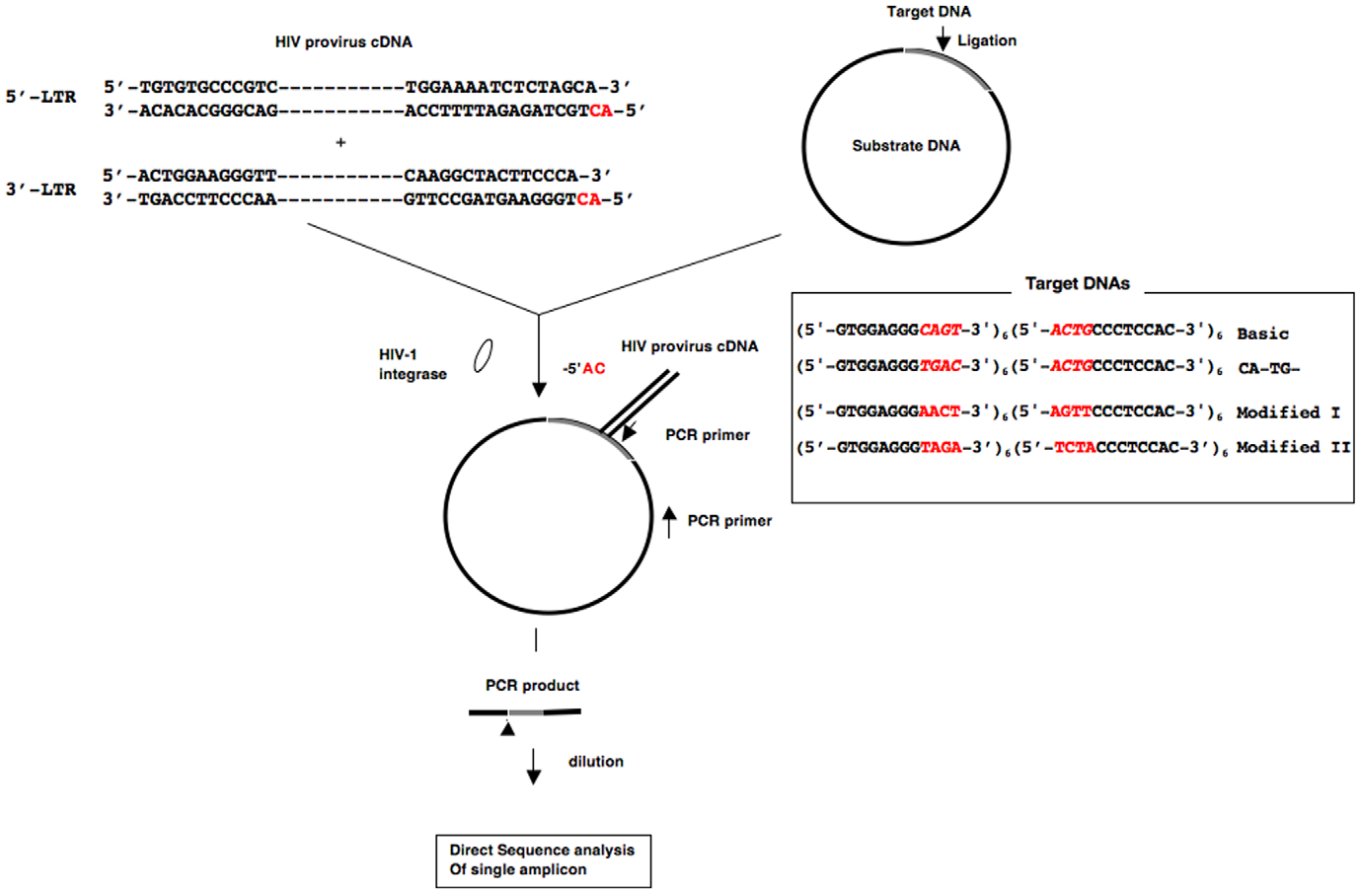
Integration into target sequence. Scheme of *in vitro* integration. Sequences of HIV-1 proviral 5′- and 3′-ends are shown. Grey segment in the circular plasmid represents the target 144-bp DNA, and the black line represents the remainder of the circular plasmid DNA used for ligation. Red letters in the HIV-1 DNA sequence represent the dinucleotides that were removed in the course of integration. Following incubation of the proviral LTR sequence DNAs with integrase, the resultant re-integrase complexes were reacted with the substrate DNA. PCR amplification was performed using primers in the proviral ends and circular DNA in the substrate DNA and the integration sites were analyzed by direct sequencing. The arrowhead in the PCR product indicates the junction between the provirus and target DNA.

We modified previously reported protocol for *in vitro* integration[9], [10], [11]. First of all, although little attention has been given to the target sequence motifs, we performed *in vitro* integration assay using the repeat sequence (5′-GTGGAGGG*CAGT*-3′)*_6_* *(5′-ACTG* *CCCTCCAC-3′*)_6_, basic sequence. *CAGT* and *ACTG*, shown in italics in red, originated from the LTR end of the HIV-1 provirus. The repeat sequence units 5′-GTGGAGGG*CAGT*-3′ *and 5′-ACTGCCCTCCAC-3′* are indicated by *x* and *y*, respectively, with the complete target sequence being *x_6_y_6_*. In addition, we designated the 6 repeat unit sequences starting from the 5′ end as *nx (n = 1, 2, 3, 4, 5, 6)* and *ny (n = 1, 2, 3, 4, 5, 6)*, respectively. We also synthesized four random 144-bp sequences designed by a random number generator and ligated them into the circular DNA in the same manner to serve as controls. In order to prevent non-specific reactions at the target DNA sequence, we ligated the target sequence DNA to circular plasmid DNA (invitrogen pCR2.1 TOPO vector) and used the whole DNA as the substrate DNA in the present assay (Fig. 1). Other modified sequences, CA-TG- and modified sequences I and II, are also listed under the basic sequence. In these modified sequences, the red letters in italics represent the four replaced nucleotides in the basic sequence.

In our protocol, the prepared HIV-1 U3 in 5′-LTR and U5 in 5′-LTR DNA were mixed and incubated with integrase prior to integration, and the prepared pre-integration complexes were then reacted with substrate DNA. During *in vivo* integration, dinucleotides at the 3′ ends of the (+) strand in the 5′-LTR and 3′-LTR are removed by integrase in the initial step prior to integration reaction[12]. Here, we used HIV-1-cDNA with dinucleotides that had already been removed. Following incubation, we performed PCR using primers for the HIV-1 U3 in 5′-LTR and U5 in 5′-LTR, and a primer for the substrate DNA consisting of the target DNA ligated with the circular DNA. This control sequence used in the assay of a co-existing modified target sequence was completely random as a result of its preparation with the use of a table of radon numbers in the absence of a palindromic or inverted repeat. The sequence motifs were calculated by GENETYX Ver10 software (Genetyx Co., Ltd., Tokyo, Japan). We prepared ten types of sequences, and the data is the average of the results.

Recombinant HIV-1 integrase was kindly provided by Dr. Yoshinaga. HIV-1 *in vitro* integration was devised as follows. First, 75 ng of U5′-LTR (long terminal repeat) sequence cDNA from HIV-1, (+) 5′-TGT GTG CCC GTC TGT TGT GTG ACT CTG GTA ACT AGA GAT CCT CAG ACC TTT TTG GTA GTG TGG AAA ATC TCT AGC A-3′ and (-) 5′-ACT GCT AGA GAT TTT CCA CAC TAC CAA AAA GGG TCT GAG GGA TCT CTA GTT ACC AGA GTC ACA CAA CAG ACG GGC ACA CA-3′, was incubated with 50 ng recombinant HIV-1 integrase in 10 µl of binding buffer for 1 h at 30°C. The binding buffer consisted of 1-0.1 mM MnCl_2_, 5 mM MnCl_2_, 80 mM glutamate potassium glutamate, 10 mM mercaptoethanol, 10% DMSO, and 35 mM MOPS (pH 7.2). Similarly, U3′-LTR sequence cDNA (75 ng) from HIV-1, (+) 5′-ACT GGA AGG GTT AAT TTA CTC CAA GCA AAG GCA AGA TAT CC TTG ATT TGT GGG TCT ATA ACA CAC AAG GCT ACT TCC CA-3′ and (-) 5′-ACTG GGA AGT AGC CTT GTG TGT TAT AGA CCC ACA AAT CAA GGA TAT CTT GCC TTT GCT TGG AGT AAA TTA ACC CTT CCAGT-3′, was incubated with the recombinant retroviral integrase. After incubation, the individual double-stranded (ds) U5′-LTR DNA was combined with the ds U3′-LTR DNA for 1 h at 30°C, and the LTR DNA further incubated with the target DNA for 1 h at 30°C. The proportion of the weight of the LTRs and target DNAs was optimalized for the prevention of a non-specific reaction and of multiple integation due to an excess of LTRs. The DNA in the buffer was purified using a QIA quick column. PCR amplification was then performed using the retroviral primers HIV-1 U5′-LTR 5′-GTG TGC CCG TCT GTT GTG TGA CTCTGG-3′, or HIV-1 U3′-LTR primer 5′-CTG GGA AGT AGC CTT GTG TGT TAT AG-3′, and a TOPO vector primer 5′-TCA CTC ATG GTT ATG GCA GC -3′ for nucleotide position 2222 in the TOPO-pCR2.1 vector. Single PCR product was analyzed by Genome Sequencer FLX System (Roche Diagnostics, Mannheim, Germany). The mean read number was 3.2x10^4^. The copy number of amplicon was quantified following identification of HIV-1-substrate DNA junction.

### Statistical analysis

An unpaired *t*-test test was calculated using SPSS software (SPSS, Chicago, IL, USA). *P* values <0.05 were considered statistically significant.

## Results

### Evaluation of *in vitro* integration efficiency and selectivity

Here, we describe the result of the *in vitro* integration assay. The amplification product is referred to below as a post-integration amplification product (PIAP). Direct sequence analysis of individual PIAPs was then performed in order to identify the integration site.

**Figure 2:**
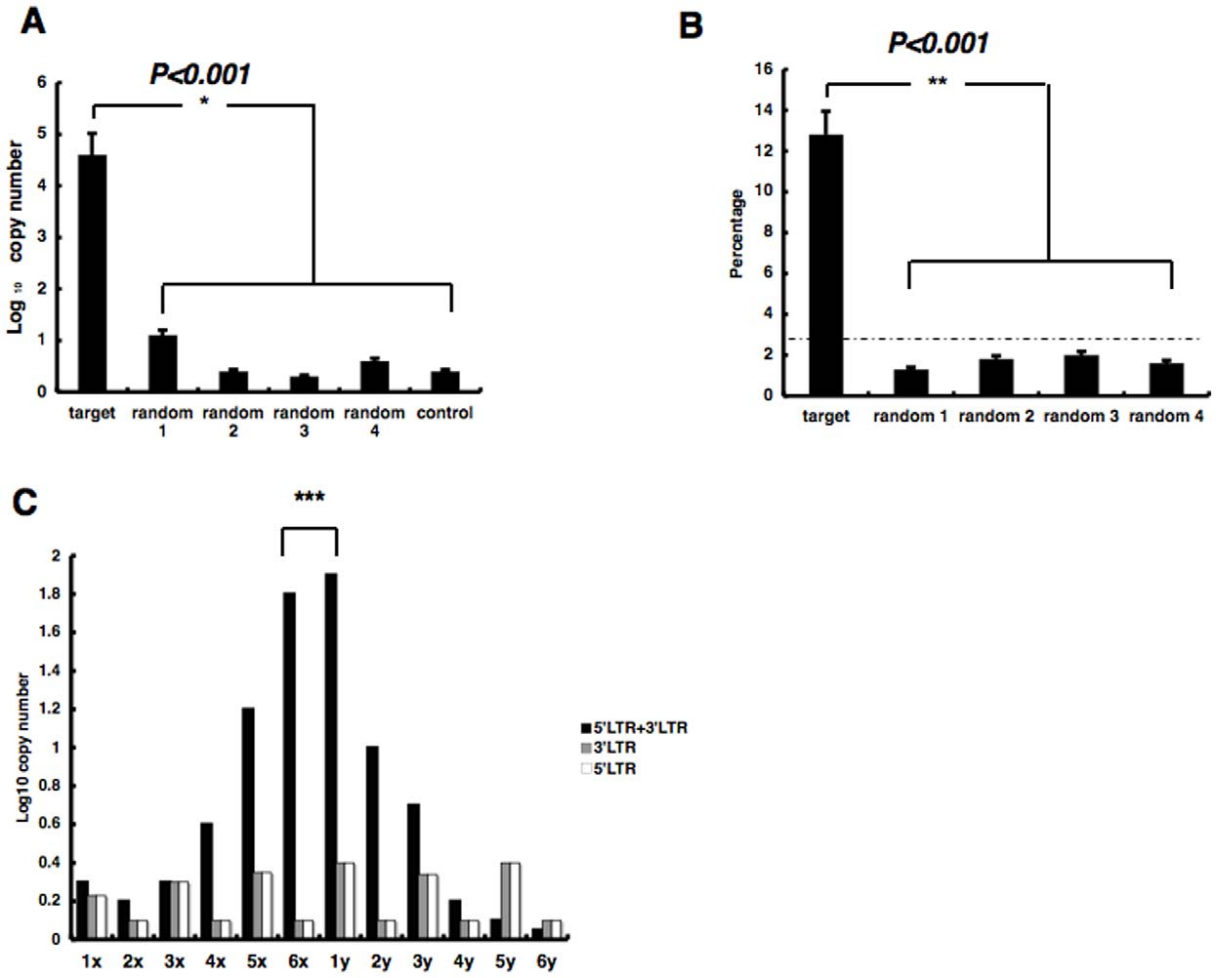
*In vitro* integration efficiency and selectivity. (A) Copy number of PCR products with using primers for the HIV-1 LTR and substrate DNA following integration into the target sequence or random sequences in the substrate DNA, referred to as post-integration amplified products (PIAP). The vertical axis represents log PIAP. Error bars indicate standard deviation (S.E.) (*P<0.001). Plasmid DNA lacking the target sequence was used as a control. (B) Percentages of PIAP copies from the integration into the target sequence or random sequences vs. the total number of PIAP. Dotted line shows the ratio when integration was thought to occur uniformly in the 4-kb substrate DNA. Error bars represent standard deviation (S.E.) (**P<0.001). (C) Number of PIAP copies from integrations into individual segments in x_6_y_6_. Vertical axis indicates log PIAP copy number. Error bars represent standard deviation (S.E.) (***P<0.001, 6x&1y vs. other segments).

The ratio of PIAP copy numbers to the total PCR amplification product reached approximately 5 times the percentage of the base length of the target 144 bp to the total DNA substrate base length of 4.1 kbp (3.5%). In contrast, we found that the ratios of the PIAP copy numbers into the random sequences of 144 bp were not significantly different from the base length percentage. The copy numbers of PIAPs arising from the target sequence were significantly greater than those when applying random sequences (Fig. 2A, B). When using random sequences 1 and 4, although a relatively higher, but not significantly, copy number of PIAP was obtained due to the greate copy number of amplicons of non-specific integration into the cloning vector sequence, the number was significantly lower than that of PIAI when using the target sequence.

On the other hand, when the integration reaction was carried out by a previous method using only the 5′ LTR or 3′ LTR sequence of HIV-1, the PIAP copy ratio was less than the base length percentage (Fig. 2B). Interestingly, even though the same sequence units (six x-segments and six y-segments) were repeated in the target sequence, we learned that high frequency integration occurs at site {6x, 1y} located at the middle of the repeat unit sequence (Fig. 2C).

### CA and GT dinucleotides were preferred for HIV-1 integration

**Figure 3:**
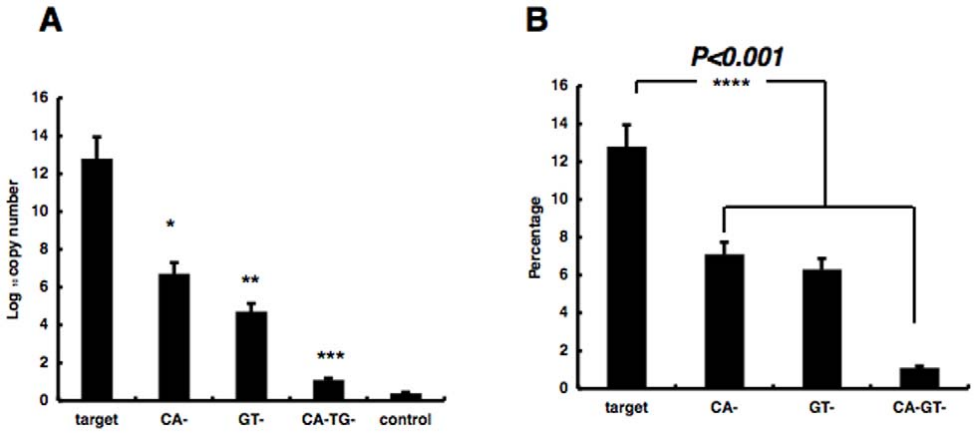
Presence of CA and TG motifs and integration ratio. (A) Number and (B) percentage of PIAP copies from integration into target sequences or sequences lacking 5′-CA-3′ or 5′-GT-3′ dinucleotide motifs (*p<0.001; target vs. CA-; **p<0.001 vs. GT- ; ***p<0.001 vs. CA-GT-; **** *p<0.*001; target vs. CA-, GT-, CA-GT-). Error bars represent standard deviation (S.E.). The sequences shown display the 6x1y segment of the target DNA. The sequence CA-TG- in Figure 1 indicates an example of CA-TG- sequences.

Next, target DNA was prepared comprising 4^4^−1 = 255 combinations without the 4 bases at the *CAGT* site on the 3′ end of unit 6x in the integration target sequence (see sequences, Fig. 3A), and PCR was carried out after insertion into circular DNA by using the primer set including the HIV-1 U5′-LTR, or the HIV-1 U3′-LTR primer and the TOPO vector primer in the TOPO-pCR2.1 vector. We took the average of the CA-, GT-, and CA-GT- PIAP for the calculation by the dividing the total copy numbers by the whole copy numbers of PCR products. The results revealed that the integration product copy number into the target sequence that contained both CA and GT was significantly greater than the copy numbers into sequences that lacked CA, GT, or both (Fig. 3A, B).

### Correlation between target sequence length and integration efficiency and selectivity

**Figure 4:**
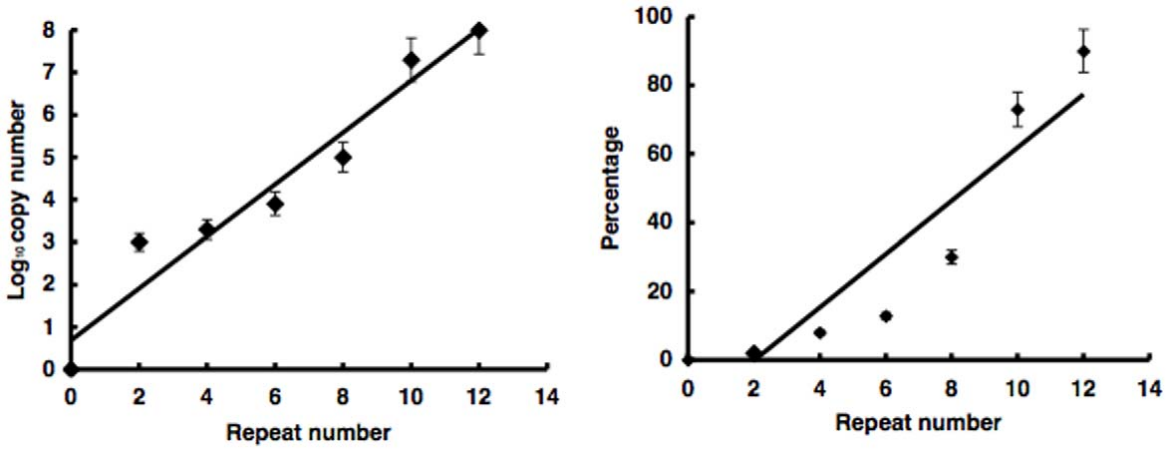
*In vitro* integration using modified or variable-length target sequences. Number of PIAP copies from integration into variable-length repeat sequences (left). Line represents a linear regression between the logarithm of copy number and number of repeated x, y units. The correlation coefficient was equivalent to 0.96. Repeat number 0 indicates that plasmid DNA was used as the substrate. Error bars represent standard deviation (S.E.). Percentage of PIAP copies from integration into the variable repeat sequences against that from integration into the whole sequence (right). Line represents linear regression between the percentage and number of repeated x, y units. The correlation coefficient was equivalent to 0.92. Error bars represent standard deviation (S.E.) (*P<0.001).

We then varied the repeat number of x- and y- segments in the target sequence and investigated PIAP copy number and ratio vs. whole PIAP products. The repeat number of the target sequence was positively correlated with the copy number and percentage (Fig. 4). The square of the correlation coefficients was 0.901 and 0.874, respectively.

### Co-existing modified target sequence DNAs interfered with integration into the original target sequence

**Figure 5:**
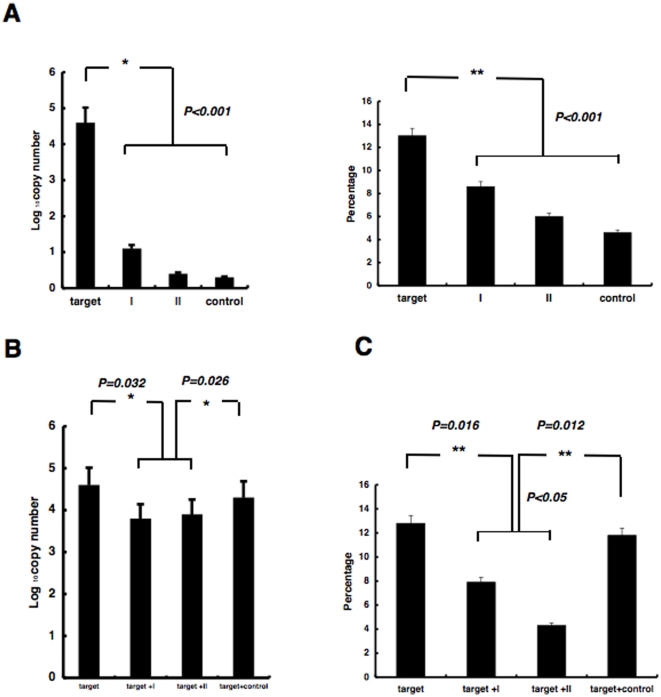
Interference effects of coexisting modified DNA sequences. (A)Number and percentage of PIAP copies from integration into the target sequence or into modified sequences I or II. Control was plasmid DNA. Error bars represent standard deviation (S.E.) (*P<0.001). (B)(C) Individual bars show logarithms of number of PIAP copies (B) and percentage of PIAP copies (C) using substrate DNA including the target sequence alone, target plus modified sequence I (left), or target plus modified sequence II (right). The amounts of target and modified sequences were equivalent. Plasmid DNA was used as a control. The percentage was calculated from the ratio of PIAP copies from integration into the target sequence against that from integration into the whole substrate DNA (*, **P<0.05). Error bars represent standard deviation (S.E.).

We then investigated whether the palindromic sequences flanking the 5′-CAGT-3′ motif increased the number of PIAP copies. We prepared two modified DNA sequences in which 5′-CA-3′ and 5′-GT-3′ were removed from the 6x segment: modified sequence I and modified sequence II (Fig. 1, modified I and II). *In vitro* integration using modified sequence I or II revealed significant reductions in the number of PIAP copies. In addition, integration selectivity was not evident when using the modified DNA sequences (*P*>0.05) (Fig. 5A).

Next, we mixed substrate DNA containing the original target sequence and substrate DNA containing modified sequence I or II in equal amounts, and examined the number and ratio of PIAP copies originating from integration into the original target sequence.

Integration into the original target sequence DNA in the substrate DNA was significantly reduced when the substrate DNA including modified sequence was mixed. In contrast, the integration was not reduced when substrate DNA including random 144 bp sequence was mixed (Fig. 5B, C).

### Correlation between concentration of manganese dichloride and integration efficiency/selectivity

**Figure 6:**
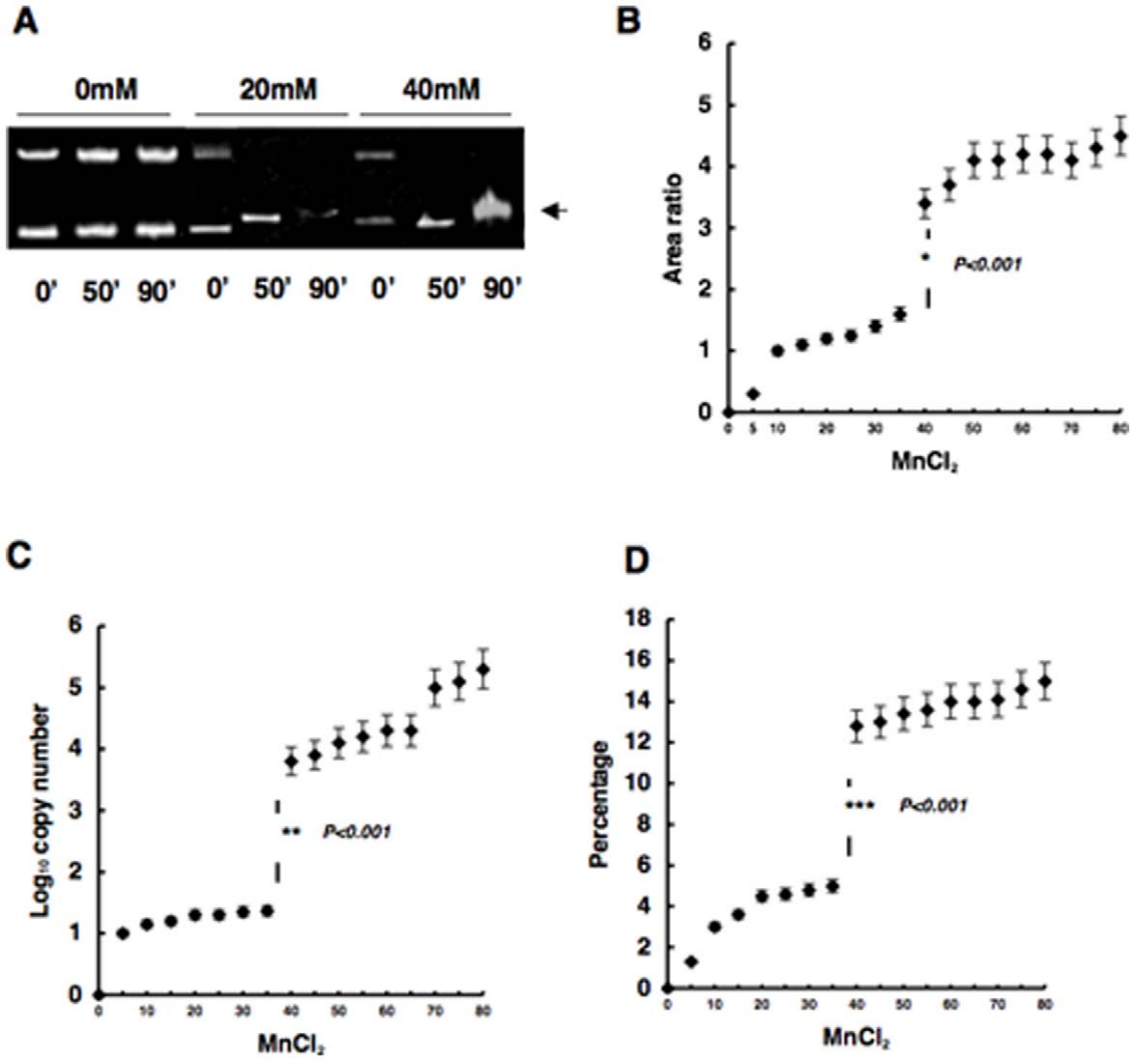
Concentration of MnCl_2_ and integration efficiency/selectivity. (A) The photo shows electrophoresis of digested substrate DNA in buffer containing 20 mM or 40 mM MnCl_2_ following incubation for 0, 50 and 90 minutes. An arrow indicates the digested 4.0-kb linearized DNA. (B) Relative area intensity of digested 4.0-kb substrate DNA after electrophoresis. Error bars represent standard deviation (S.E.). The graph shows the area ratio of the digested DNA band on electrophoresis in (A). Error bars represent standard deviation (S.E.). (C) Number of PIAP copies in buffer containing various concentrations of MnCl_2_. Error bars represent standard deviation (S.E.). (D) Percentage of PIAP copies in buffer containing various concentrations of MnCl_2_. Error bars represent standard deviation (S.E.).

We digested circular DNA in buffer containing various concentrations of manganese dichloride and measured the band intensity of linearized DNA following electrophoresis. On the basis of our observation, both the upper and lower fragments in the absence of MnCl_2_ were probably identical to the conformational isomer of undigested circular DNA that was was comprised of the plasmid sequence DNA and the target DNA. In the presence of MnCl_2_, the apparent fragment appeared, and this new fragment was digested by the linear DNA. Fluctuations in the mobility of digested DNA increased significantly when the concentration of MnCl_2_ exceeded 40 mM (Fig. 6A). Moreover, to quantitatively evaluate the fluctuations in mobility, we calculated the area of electrophoresed DNA bands by normalizing the area of electrophoresed DNA bands that were digested in buffer containing 10 mM of MnCl_2_ to 1.0. The relative area discontinuously increased when the concentration of MnCl_2_ exceeded 40 mM, indicating that higher concentrations of MnCl_2_ induced heterogeneity in the secondary structure of substrate DNA(Fig. 6B)(*P<0.001).

Similarly, the copy number of PIAP from integration into the target sequence DNA was found to increase significantly when the concentration of MnCl_2_ exceeded 40 mM (Fig. 6C)(**P<0.001). Moreover, the ratio of copy number of PIAP from integration into the target sequence DNA to the total copy number of PIAP was found to increase significantly when the concentration of MnCl_2_ exceeded 40 mM (Fig. 6D) (***P<0.001).

## Discussion

The finding shown in Fig. 2A, B reveals that the integration rate into the target sequence used in this study was significantly greater than the integration rate into the random sequences. If the integration occurred at equivalent frequency in the whole target sequence, the percentage was nearly the base length ratio, e.g., 144 bp to 144 plus 3894 base. Of course, the percentage was influenced by the PCR primer setting, the value was one of the standards use to evaluate the integration selectivity.

Thus, we showed that HIV-1 integration favors a specified sequence at least. Such data in Fig. 2A–C and Fig. 3 clearly show that both the nucleotides serving as the reaction target and their adjacent segments affect reaction efficiency. In Fig. 2A and B, the ratio of the PIAP copy numbers into the random sequences of 144 bp was lower than that predicted for at least the random sequences 1 and 4. There were probably differences in the frequency of appearance of 5′-CA and 5′-TG in the sequence. In random sequences 1 and 4, these dinucleotide motifs appear 5 and 7 times, individually, i.e., 10 and 9 times less than those in random sequences 2 and 3. The lower frequence probably influences the copy number, eg., integration efficiency. This data is suggestive of the following evaluation shown in Fig. 3. In addition, data shown in Fig. 2C demonstrated that the combined presence of the 5′ LTR terminus and the 3′ LTR terminus promotes integration into the target sequence. This combination is found to be critical in *in vitro* integration, suggesting a possibility that similar co-operation of the 5′ LTR terminus and the 3′ LTR terminus contributes to *in vivo* integration. In Fig. 3, we showed that 5′-CA and 5′-GT are apparently favored in *in vitro* integration. As Yoshinaga et al. already suggested, the identical dinucleotide motifs are observed in the LTR and are ctirical motifs for integration. Therefore, we supposed that HIV-1 pre-integration complex including LTR favors 5′-CA and 5′-GT in target sequences that are complementary to the dinucleotide.

The data of close correlation between integration efficiency/selectivity with the repeat number shown in Fig. 4 suggest that the flanking sequences actually influences reaction efficiency in addition to target nucleotides. Moreover, the whole repeat sequence or secondary structure may be target of integration.

Especially, our findings of interference by sequences similar to the target DNA sequence suggest that such effects actually interfere with integration selectivity (Fig. 5). The modified DNA can act as a decoy for the target DNA.

In the present study, integration efficiency and selectivity were highly sensitive to MnCl_2_ concentration in the reaction buffer. In particular, when increasing MnCl_2_ from 30 mM to 40 mM, the integration efficiency and selectivity increased significantly. Similarly, fluctuations in electrophoretic mobility of substrate DNA also increased. This suggests that there is a threshold concentration of MnCl_2_ for *in vitro* integration, probably because MnCl_2_ induces instabilities in secondary structure and phase transition of the host DNA strand may occur [13], [14]. As presented in Fig. 6B, the change remained in the fluctuation of electromobility as the MnCl2 concentration became higher. Probably, target DNA cannot generate the specified stable conformation under this condition. Taken together with these data and those shown in Fig. 4, we supposed that there are close correlations between structural changes in substrate DNA, and integration selectivity and efficiency. We have been studying *in vitro* integration using magnesium chloride because this salt is more appropriate for the regeneration of *in vivo* integration. We will report the result elsewhere.

In actual integration into the host genome, numerous DNA-binding proteins and metal ions regulate the reaction in a complex manner. Therefore, the present data cannot be immediately applied to *in vivo* systems and further investigation using cell culture systems are necessary. However, this report is expected to facilitate understanding of the pathogenicity of HIV-1.

## References

[1] Grinsztejn B, Nguyen BY, Katlama C, Gatell JM, Lazzarin A (2007). Safety and efficacy of the HIV-1 integrase inhibitor raltegravir (MK-0518) in treatment-experienced patients with multidrug-resistant virus: a phase II randomised controlled trial.. Lancet.

[2] Christ F, Voet A, Marchand A, Nicolet S, Desimmie BA (2010). Rational design of small-molecule inhibitors of the LEDGF/p75-integrase interaction and HIV replication.. Nat Chem Biol.

[3] Bar-Magen T, Sloan RD, Donahue DA, Kuhl BD, Zabeida A (2010). Identification of Novel Mutations Responsible for Resistance to MK-2048, a Second-Generation HIV-1 Integrase inhibitor.. J Virol.

[4] Schroder AR, Shinn P, Chen H, Berry C, Ecker JR (2002). HIV-1 integration in the human genome favors active genes and local hotspots.. Cell.

[5] Wu X, Li Y, Crise B, Burgess SM (2003). Transcription start regions in the human genome are favored targets for MLV integration.. Science.

[6] Holman AG, Coffin JM (2005). Symmetrical base preferences surrounding HIV-1, avian sarcoma/leukosis virus, and murine leukemia virus integration sites.. Proc Natl Acad Sci U S A.

[7] Mitchell RS, Beitzel BF, Schroder ARW, Shinn P, Chen HM (2004). Retroviral DNA integration: ASLV, HIV, and MLV show distinct target site preferences.. Plos Biology.

[8] Verdin E (1991). Dnase I-Hypersensitive Sites Are Associated with Both Long Terminal Repeats and with the Intragenic Enhancer of Integrated Human-Immunodeficiency-Virus Type-1.. Journal of Virology.

[9] Yoshinaga T, Fujiwara T (1995). Different Roles of Bases within the Integration Signal Sequence of Human-Immunodeficiency-Virus Type-1 in-Vitro.. Journal of Virology.

[10] Farnet CM, Haseltine WA (1990). Integration of human immunodeficiency virus type 1 DNA in vitro.. Proc Natl Acad Sci U S A.

[11] Kitamura Y, Lee YM, Coffin JM (1992). Nonrandom integration of retroviral DNA in vitro: Effect of CpG methylation.. Proc Natl Acad Sci U S A.

[12] Vink C, Yeheskiely E, van der Marel GA, van Boom JH, Plasterk RH (1991). Site-specific hydrolysis and alcoholysis of human immunodeficiency virus DNA termini mediated by the viral integrase protein.. Nucleic Acids Res.

[13] Ueda M, Yoshikawa K (1996). Phase transition and phase segregation in a single double-stranded DNA molecule.. Physical Review Letters.

[14] Iwaki T, Makita N, Yoshikawa K (2008). Folding transition of a single semiflexible polyelectrolyte chain through toroidal bundling of loop structures.. Journal of Chemical Physics.

